# Genomic heterozygosity is associated with a lower risk of osteoarthritis

**DOI:** 10.1186/s12864-024-10015-9

**Published:** 2024-01-20

**Authors:** Robert Gill, Ming Liu, Guang Sun, Andrew Furey, Tim Spector, Proton Rahman, Guangju Zhai

**Affiliations:** 1 Human Genetics and Genomics, Division of Biomedical Sciences, Faculty of Medicine, Memorial University of Newfoundland, St. John’s, NL A1B 3V6 Canada; 2 Discipline of Medicine, Faculty of Medicine, Memorial University of Newfoundland, St. John’s, NL Canada; 3 Division of Orthopaedic Surgery, Faculty of Medicine, Memorial University of Newfoundland; Office of the Premier, Government of Newfoundland & Labrador, St. John’s, NL Canada; 4 Department of Twin Research & Genetic Epidemiology, Faculty of Life Sciences & Medicine, King’s College London, London, UK

**Keywords:** Knee Osteoarthritis, Hip Osteoarthritis, Heterozygosity, Genomics

## Abstract

**Background:**

Genomic heterozygosity has been shown to confer a health advantage in humans and play a protective role in complex diseases. Given osteoarthritis (OA) is a highly polygenic disease, we set out to determine if an association exists between OA and genomic heterozygosity.

**Results:**

End-stage knee and hip OA patients and healthy controls were recruited from the Newfoundland and Labrador (NL) population. The Arthritis Research UK Osteoarthritis Genetics (arcOGEN) consortium database was utilized as a replication cohort. DNA was extracted from blood samples and genotyped. Individual rates of observed heterozygosity (HetRate) and heterozygosity excess (HetExcess) relative to the expected were mathematically derived, and standardized to a z-score. Logistic regression modeling was used to examine the association between OA and HetRate or HetExcess. A total of 559 knee and hip OA patients (mean age 66.5 years, body mass index (BMI) 33.7 kg/m^2^, and 55% females) and 118 healthy controls (mean age 56.4 years, BMI 29.5 kg/m^2^, and 59% female) were included in the NL cohort analysis. We found that OA had an inverse relationship with HetRate and HetExcess with odds ratios of 0.64 (95% CI: 0.45–0.91) and 0.65 (95% CI: 0.45–0.93) per standard deviation (SD), respectively. The arcOGEN data included 2,019 end-stage knee and hip OA patients and 2,029 healthy controls, validating our findings with HetRate and HetExcess odds ratios of 0.60 (95% CI: 0.56–0.64) and 0.44 (95% CI: 0.40–0.47) per SD, respectively.

**Conclusions:**

Our results are the first to clearly show evidence, from two separate cohorts, that reduced genomic heterozygosity confers a risk for the future development of OA.

## Background

Osteoarthritis (OA) is a chronic heterogeneous disease affecting multiple joint tissues and impacting over 500 million people worldwide [1]. It is characterized by a gradual loss of articular cartilage and subchondral bone changes that typically present clinically in patients as joint pain and functional limitations. Despite its high prevalence, its treatment options remain limited, with early therapies aimed at pain management, and end-stage disease treated with total joint replacement. The development of OA is multifactorial, with environmental, lifestyle, and genetic factors contributing to its progression [2, 3]. Further, OA is a highly polygenic disease and its genetic predisposition is still under heavy investigation with over 100 genetic loci found to date [4].

Genetic diversity within *Homo sapiens* has played a significant role in our survival and evolution. Literature across multiple species, including humans, supports the dogma that increased genetic diversity improves a species’ fitness [5]. From an evolutionary point of view, this diversity has been developed through breeding between genetically diverse populations, creating genetically diverse offspring. Offspring that inherit genetic variants which are favourable to their survival subsequently pass these variants on to their offspring [6, 7]. This association is termed the heterozygosity-fitness correlation, for which the underlying mechanism is still profoundly debated. A common thread among proposed theories is that the more heterogeneity within an individual’s genome, the greater fitness that individual inherits [5]. A classic example is the Major Histocompatibility Complex (MHC), where more heterozygosity in this genomic region subsequently results in increased antibody diversity and a more robust immune system [8].

With the outburst of genome-wide sequencing, other labs have shown that genome-wide heterozygosity plays a crucial role in human health. Multiple studies have demonstrated that higher heterozygosity rates are interconnected with improved long-term health outcomes and potentially confer healthier aging [9, 10]. Given that prior literature has uncovered a link between heterozygosity and chronic diseases, we undertook this study to investigate if this relationship also applies to OA. In this study, we hypothesized that higher heterozygosity would demonstrate a protective role against hip and knee OA compared to disease-free controls derived from the same population.

## Results

### Characteristics of the NL cohort

 This study included 559 OA patients (172 hip, 387 knee) and 118 controls. The OA group comprised of patients that required a total joint replacement, an indicator of end-stage disease. OA patients were found to be older (*p* < 0.0005) and have a higher average body mass index (BMI) (*p* < 0.0005) than non-OA controls, while there was no statistically significant difference in sex between OA and controls (Table 1). Additionally, within the OA group, knee OA patients were found to have a higher BMI than hip OA (*p* < 0.001), but sex and age did not statistically differ between the two joint groups (Table 1). A statistically significant correlation was noted between age, BMI, and heterozygosity (Table 2). Thus, age, sex, and BMI were adjusted for in the subsequent logistic regression models.

**Table 1:** The characteristics of participants in the NFOAS cohort

**Demographic**	**Osteoarthritis (*n* = 559)**		*P*-value^*^	**Controls (*n* = 118)**	*P*-value*
	**Hip**	**Knee**			
**Mean Age Years (± SD)**	66.5 (± 8.7)			56.4 (± 8.9)	< 0.0005
	67.4 (± 10.0)	66.0 (± 7.9)	0.086	N/A	
**Sex (Female %)**	55			59	0.401
	51	57	0.190	N/A	
**Mean BMI (kg/m**^**2**^**, ± SD)**	33.7 (± 6.8)			29.5 (± 4.9)	< 0.0005
	31.1 (± 6.2)	34.9 (± 6.7)	< 0.001	N/A	

Hip *n* = 172, Knee *n* = 387

*Two-sided student t-test

**Table 2:** Pearson correlation analysis for age, BMI and heterozygosity rates in NFOAS cohort

	SNP MAF > 0.01		SNP MAF > 0.1	
**Variable**	HetRate	HetExcess	HetRate	HetExcess
Age	-0.089^*^	-0.080^*^	-0.088^*^	-0.077^*^
BMI	0.091^*^	0.096^*^	0.090^*^	0.092^*^

Age *n* = 679, BMI *n* = 677

^*^ Correlation significant at *p* < 0.05 (two-tailed)

### Heterozygosity rates and OA in the NL cohort

**Fig. 1:**
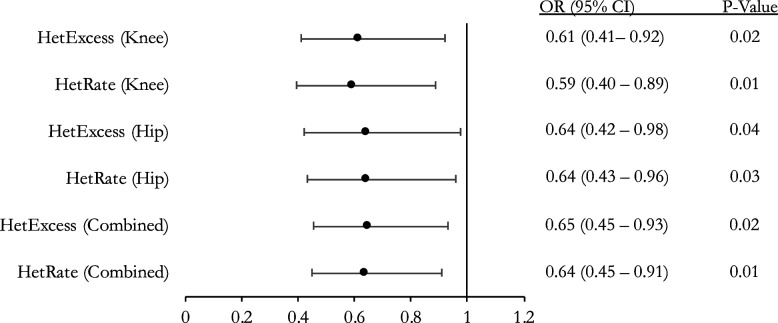
Association between heterozygosity and OA for NFOAS cohort (SNP MAF > 0.01). Multivariable logistic regression for heterozygosity rate with outcome set to OA status. Odds ratio (OR), 95% confidence interval (95% CI), and *p*-value with adjustments for age, sex, and BMI

Multivariable logistic regression with adjustments for age, sex, and BMI was performed with OA status as the dependent variable to evaluate the relationship between OA and heterozygosity. Single nucleotide polymorphisms (SNPs) with minor allele frequency (MAF) < 0.01 were excluded from the final analysis as they are less common variants and more likely to be specific to the individual population. First, we examined the relationship between OA and observed heterozygosity (HetRate) and found an inverse relationship with an odds ratio (OR) of 0.64 (*p* = 0.01; Fig. 1). To determine if this relationship was specific to one joint subgroup, we performed the analyses for hip and knee OA separately. These analyses demonstrated a statistically significant inverse relationship between OA status and HetRate for hip and knee OA with a similar effect size, an OR of 0.64 and 0.59, respectively (Fig. 1).

As observed heterozygosity rate is influenced by allele frequencies, we next performed multivariable logistic regressions with heterozygote excess (HetExcess), as this variable is independent of allele frequency. Our results showed an inverse relationship between HetExcess and OA status with an OR of 0.65 (*p* = 0.02, Fig. 1). Separate analyses for hip and knee OA exhibited similar statistical significance, with an OR of 0.64 and 0.61, respectively (Fig. 1).

**Fig. 2:**
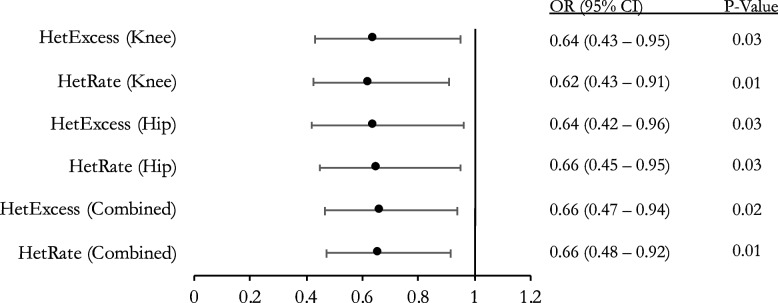
Association between heterozygosity and OA for NFOAS cohort (SNP MAF > 0.1). Multivariable logistic regression for heterozygosity rate with outcome set to OA status. Odds ratio (OR), 95% confidence interval (95% CI), and *p*-value with adjustments for age, sex, and BMI

Next, we explored if this association would remain statistically significant for more common genetic variants in our population. Therefore, we further restricted the SNP MAF to greater than 0.1. We repeated the previous regression analyses with this new dataset and demonstrated that the inverse relationship between OA status and heterozygosity remained statistically significant. This was found for all comparisons and demonstrated a similar effect size to that of SNPs with MAF > 0.01 (Fig. 2).

Further, we repeated the regression analyses with an additional adjustment for diabetes – one of the comorbidities that we had data on in our cohort. All the observed associations between OA and heterozygosity remained statistically significant (*p* < 0.05).

### Heterozygosity rates and OA in the replication cohort

**Fig. 3:**
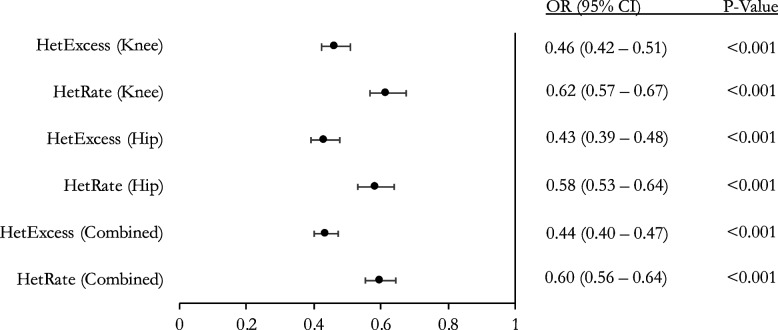
Association between heterozygosity and OA for arcOGEN cohort (SNP MAF > 0.01). Logistic regression for heterozygosity rate with outcome set to OA status. Odds ratio (OR), 95% confidence interval (95% CI), and *p*-value

**Fig. 4:**
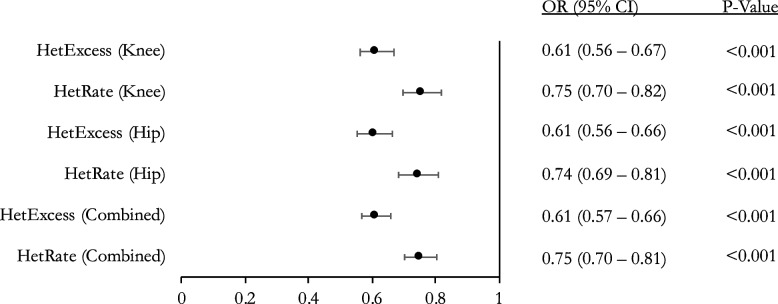
Association between heterozygosity and OA for arcOGEN cohort (SNP MAF > 0.1). Logistic regression for heterozygosity rate with outcome set to OA status. Odds ratio (OR), 95% confidence interval (95% CI), and *p*-value

To replicate and validate our results in an independent population, we utilized the Arthritis Research UK Osteoarthritis Genetics (arcOGEN) genomic database. This is a UK based cohort with 2019 OA patients and 2029 OA-free controls. A simple logistic regression was performed with OA status as the dependent variable to evaluate the relationship between OA and heterozygosity. The arcOGEN analyses validated our results by further showing an inverse relationship between OA and heterozygosity across all analyses. At SNP MAF > 0.01, HetRate and HetExcess ORs were 0.60 (*p* =  < 0.001) and 0.44 (*p* =  < 0.001) per standard deviation (SD), respectively (Fig. 3). Subgroup analysis of hip and knee OA separately showed a consistent statistically significant relationship between OA and heterozygosity (Fig. 3). At SNP MAF > 0.1, HetRate and HetExcess ORs were 0.75 (*p* =  < 0.001) and 0.61 (*p* =  < 0.001) per SD, respectively (Fig. 4). Subgroup analysis of hip and knee OA separately showed a consistent statistically significant relationship between OA and heterozygosity (Fig. 4).

## Discussion

The genetics of OA continues to be an actively investigated field of research, with an increasing number of genomic variants being discovered. In this study, we took a unique approach by examining the association between genomic heterozygosity and OA. Previous studies have found that increased heterozygosity is associated with healthier aging, a lower risk of death, and lower health parameters such as blood pressure and cholesterol [9–11]. Our data showed a statistically significant but small correlation between genomic heterozygosity and age/BMI. The negative correlation with age was most likely due to the fact that the OA patients were older than the controls in our cohort whereas the positive correlation with BMI might be explained by the evolutionary advantage of fat storage and lower energy expenditure. However, to the best of our knowledge, this was the first study to provide evidence that heterozygosity rates are correlated to the risk of end-stage OA. Indeed, we found that both observed heterozygosity and heterozygosity excess were statistically significantly lower in OA patients across all comparisons in our cohort. Additionally, we were able to validate these finding in a large independent UK cohort. These findings advance the field of OA research by further strengthening the known role of genetics in OA development. It also provides evidence that genetic heterozygosity plays an important role in patients being predisposed to chronic diseases such as OA.

Furthermore, this relationship remained significant for SNPs with MAF > 0.1, signifying that the association with heterozygosity also exists for common alleles. These findings indicate that the mechanism behind the protective role seen for increased heterozygosity is not due to purifying selection against deleterious recessive alleles. Xu et al*.* demonstrated similar results for the association between healthier aging and increased heterozygosity [9]. Their study categorized SNPs by MAF and found that SNPs with more common allele frequencies had a similar significance to those with lower frequencies. They argued this refutes the notion that increased heterozygosity provides its benefit through compensation for deleterious recessive alleles [9]. Instead, it supports the mechanism of heterozygote advantage or overdominance [9]. The heterozygosity-fitness correlation in our study is likely under-pined by the same mechanism.

While an abundance of genetic variants associated with OA have been detected, research is limited by the fact that the majority of these variants are found within non-coding regions of the genome and have a small effect size [4, 12]. This has led to the prevailing theory that the accumulation of multiple risk alleles contributes to the predisposition of OA later in life and that a threshold must exist that tips an individual into OA. This theory also explains the diversity seen in OA patients, since countless combinations of different genotypic variants could contribute to the phenotypes of OA. This polygenetic nature of OA has made early detection and targeted treatments challenging to establish. In this study, we provided a framework to use genomic heterozygosity to detect individuals at risk of OA, which has the potential to enable earlier interventions. Not a novel idea since Lacaze et al*.* and other groups have demonstrated that genomic risk scores, calculated using known genetic risk variants, can predict an individual’s risk of developing advanced hip and knee OA [13, 14]. A recent large meta-analysis in Cell also demonstrated that polygenic risk scores have predictive power for the odds of developing OA [4]. However, these models are limited because they only consider genetic variants that have already been discovered. Therefore, it cannot account for OA variants that have yet to be revealed and is only as powerful as the most current literatures on risk loci. While polygenic risk scores and genomic heterozygosity both use differences within the genome to predict an individual’s risk of OA, they are very different approaches. This is the first study to show that genomic heterozygosity has an association with OA and therefore the predict power of this approach requires further research.

This study's strengths include a well phenotyped NL cohort from a similar ethnic background for both cases and controls. The island of Newfoundland is a young founder population with a high degree of genetic and cultural homogeneity, providing a rich resource for investigating complex genetic diseases such as OA [15, 16]. However, while the limited diversity of this population enables a higher signal-to-noise ratio, the same characteristic limits our ability to generalize results to other populations. Therefore, the replication cohort allows us to further strengthen our findings through replication in an independent European cohort. Still we are limited in extrapolating these findings to other populations with different ethnic backgrounds since NL’s ancestry is primarily European in origin [16]. Also, we only included end-stage hip and knee OA patients in this study, and it is well known that joint-specific genetic patterns exist for OA sites [4]. Therefore, we cannot extrapolate these findings to other joint sites impacted by OA such as hand and spinal OA. As well, these findings cannot be generalized to mild or moderate OA patients. Controls were younger than OA patients, and some of them may develop OA later on, which might potentially bias our findings. However, this misclassification can only bias the results towards null, thus, the actual association might be even stronger than what we observed. Additionally, we did not have data on other comorbidities and factors such as socioeconomic status and genu varum/valgum which might have confounded our findings. However, this is less likely as most of those comorbidities do not have strong evidence to support their associated with OA as per a recent review on this topic [17]. Additionally, this is supported by our further analysis with adjustment for diabetes in the NL cohort that did not alter the results. Most importantly, our NL cohort findings were replicated in an independent cohort collected from a different geographic location, indicating the robustness of the findings.

In summary, these results were the first to show a clear association between genomic heterozygosity and the risk of OA. In particular, our group provides evidence from two separate population cohorts that reduced genomic heterozygosity confers a risk for the future development of OA. This supports our original hypothesis that higher heterozygosity would demonstrate protection against hip and knee OA. Additionally, we propose that the likely mechanism behind our findings is due to heterozygote advantage or overdominance. Future work is required to validate this association in other large OA genomic databases and for OA sites other than the hip and knee. Specifically, further research on non-weight bearing joint such as hand and shoulder, where BMI has less influence, is required. Furthermore, while this study provides evidence for a link between OA and heterozygosity, it does not provide any quantitative measures for the level of heterozygosity that confers a protective effect. Therefore, future predictive modelling for heterozygosity with large OA cohorts that include different stage of disease is required to delineate this model's predictive efficacy further. Once a predictive model is developed then prospective studies can determine if genomic heterozygosity can effectively predict individuals that will develop OA. Once validated, this can lead to early detection and earlier interventions for individuals at risk of OA.

## Method

### Study participants

The initial analysis utilized two cohorts with participants’ demographic and genome-wide genotype data, referred to as the NL cohort. One contained known end-stage OA patients, and the other healthy OA-free controls, both derived from the population of Newfoundland & Labrador, Canada. Self-reported OA-free controls were selected from the Complex Diseases in the Newfoundland Population; Environment and Genetics (CODING) study and OA cases included were from the Newfoundland Osteoarthritis Study (NFOAS). Inclusion criteria for the CODING study has been previously published and included: 1) a third generation or greater Newfoundlander, 2) age of 20–79 years old at the time of recruitment, 3) no serious metabolic, cardiovascular, or endocrine disorder and, 4) not pregnant at the time of the study [18–20]. The characteristics of the NFOAS study have been previously published by our group and included 1086 patients who underwent total knee or hip replacement between November 2011 and September 2017 largely due to OA [21, 22]. Only patients with a primary diagnosis of OA, as defined by the American Rheumatology College’s criteria and judgment of an attending orthopedic surgeon, were included in this study. Both studies were approved by the Health Research Ethics Authority of Newfoundland and Labrador (HREB; NFOAS—# 2011.311 & CODING—#10.33), and written informed consent was obtained from all participants. All methods were performed in accordance with the relevant regulations of this authority.

Genome-wide genotype data from the arcOGEN consortium database was utilized as a replication cohort [23]. We used data on 2029 female controls and 2019 female end-stage OA (hip or knee) patients in our replication because only female controls were available to us. The inclusion and exclusion criteria of the arcOGEN has been previously published [23]. All OA cases were unrelated and were European in origin, while controls were from the TwinsUK cohort [24]. Diagnosis of primary OA (hip or knee) was defined by a clinical level of disease that required total joint replacement. Patients with concurrent diagnoses of hip and knee OA were included in both the hip and knee subgroup analyses. All participants gave informed written consent and the study protocols were reviewed and approved by the Oxfordshire Research Ethics Committee with reference 07/H0606/150.

### Patient and public involvement

Patients were involved in our study as study participants, but neither they nor the public were involved in any of the stages of our research.

### Genome-wide genotyping

For the NL cohort, DNA was extracted from whole blood samples collected from all study participants by using standard protocol. Genome-wide genotyping was done by using either the Illumina Human Omni2.5–8 (Illumina, San Diego, CA, USA) microarray platform which genotypes 2.5 million SNPs or the Human Global Diversity Array (Illumina, San Diego, CA, USA) which genotypes 1.9 million SNPs at The Centre for Applied Genomics in Toronto (http://www.tcag.ca/index.html). The genotyped data underwent a strict quality control (QC) procedure including removing SNPs with MAF < 0.01, missing genotype rate > 5%, and out of Hardy–Weinberg Equilibrium, as well as removing individuals with discordant sex information and duplicates. Population structure was examined with the 1000 Genomes Project data as references and outliers were removed. QCed genotype data were then imputed against 1000 Genome Project phase 3 data by Sanger Imputation Server (https://imputation.sanger.ac.uk/?about=1) which generated data on a total of 81,706,022 SNPs across the entire genome. The imputed data underwent QC procedures as described above and were then used for subsequent analyses.

For the arcOGEN cohort, genome-wide genotyping was done by Illumina Human 610-Quad BeadChips (Illimina, San Diego, CA, USA) with a protocol previously described [23]. The same QC procedures as the NL cohort was performed on the directly genotyped data and then imputed by the Sanger Imputation Server with the same parameters as our discovery cohort.

### Heterozygosity

**Fig. 5:**
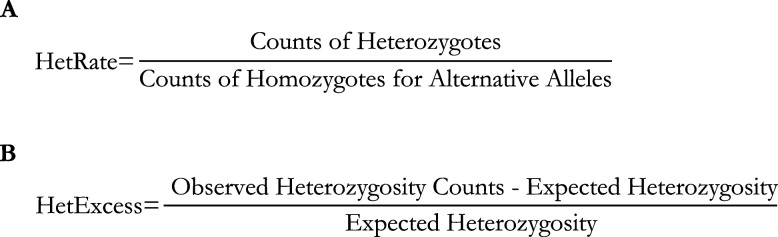
Equation utilized to calculate observed heterozygosity rate and heterozygosity excess

The total number of heterozygote genotypes, the total number of homozygote genotypes for alternative alleles, and the expected heterozygosity rates from the imputed genomic data were calculated using Plink Version 1.9 [25]. HetRate was mathematically defined as the total number of heterozygote genotypes divided by the total number of homozygote genotypes for alternative alleles (Fig. 5A). HetExcess was mathematically defined as the observed heterozygosity subtract the expected heterozygosity and then divided by the expected heterozygosity (Fig. 5B). Both HetRate and HetExcess were then standardized to z-scores prior to statistical analysis. Both HetRate and HetExcess were calculated twice, one with all SNPs with MAF > 0.01 and the other with SNPs with MAF > 0.1. There were various approaches used in the literature to represent heterozygosity. We chose these two methods because the observed heterozygosity rate provides an assessment of the current genetic diversity within a population whereas heterozygosity excess can offer insight into the historical and demographic processes that have shaped this diversity.

### Statistical analysis

For the NL cohort multivariable logistic regression modeling was utilized to investigate the association between heterozygosity and OA. Given age and BMI were found to correlate with heterozygosity, they were included in the regression along with sex to control for potential confounding effects. For the replication cohort simple logistic regression modeling was utilized to investigate the association between heterozygosity and OA. For this cohort all participants were female and data on age and BMI were unavailable. Separate regressions were run for HetRate and HetExcess, at the level of SNP MAF > 0.01 and > 0.1, respectively. Regressions were repeated to examine the association of heterozygosity with hip and knee OA separately. ORs and their 95% confidence intervals were extracted from each logistic analysis to illustrate the correlation between OA and genetic heterozygosity. ORs were calculated as the odds of developing OA for each SD increase in HetRate/HetExcess. An OR of < 1 in our model signifies that as genomic heterozygosity increases the odds of developing OA decrease. The significance level for all regression analyses was defined by an alpha level of 0.05 and all analyses were performed using statistical software SPSS version 28.0.1.0.

## Data Availability

The datasets used and/or analysed during the current study are available from the corresponding author on reasonable request.
